# Ubiquitin-mediated signalling and Paget's disease of bone

**DOI:** 10.1186/1471-2091-8-S1-S5

**Published:** 2007-11-22

**Authors:** Robert Layfield, Barry Shaw

**Affiliations:** 1 School of Biomedical Sciences, University of Nottingham Medical School, Nottingham NG7 2UH, UK

## Abstract

Multiple steps in the RANK-NF-κB signalling pathway are regulated by ubiquitylation. Mutations affecting different components of this pathway, including the ubiquitin binding p62 signalling adapter protein, are found in patients with Paget's disease of bone or related syndromes. Here, we review the molecular defects and potential disease mechanisms in these conditions and conclude that the mutations may confer a common increased sensitivity of osteoclasts to cytokines, resulting in disordered NF-κB-dependent osteoclast function. Modulation of the osteoclast RANK-NF-κB signalling axis may represent a viable therapeutic strategy for Paget's disease and other conditions where excessive bone resorption or remodelling is a feature.

**Publication history:** Republished from Current BioData's Targeted Proteins database (TPdb; ).

## Role of ubiquitin and the UPS in Paget's disease of bone and related disorders

### Background

Vertebrates regulate bone mass by the process of remodelling, which is controlled by the opposing actions of bone-forming osteoblasts and bone-resorbing osteoclasts. Increased osteoclast-mediated bone resorption is a consistent feature of skeletal disorders including osteoporosis, rheumatoid arthritis and Paget's disease of bone (PDB). This article focuses on the role of ubiquitin and the ubiquitin proteasome system (UPS) in the latter condition, PDB [MIM 167250, 602080], which is common in Caucasian populations, affecting up to 3% of individuals aged over 55 years [1]. In this case, increased resorptive activity of Pagetic osteoclasts results in secondary increases in osteoblast activity, causing focal increases in bone turnover. The new bone formed tends to be of abnormal architecture, accounting for symptoms such as bone pain, skeletal deformity, deafness, neurological complications and susceptibility to pathological fractures, which are seen in up to one third of patients [2]. Osteosarcoma is a rare complication of PDB, with the majority of adulthood osteosarcomas occurring in patients with this disease [3]. Several PDB-related syndromes show overlapping and/or often more severe symptoms or earlier onset than PDB, including familial expansile osteolysis (FEO [MIM 174810]), expansile skeletal hyperphosphatasia (ESH), early-onset familial PDB, and juvenile hyperphosphatasia (also known as juvenile PDB). Other disorders in which characteristic Pagetic changes also feature include inclusion body myopathy associated with PDB and frontotemporal dementia (IBMPFD) [4,5].

### Molecular defects in PDB and related syndromes

**Figure 1:**
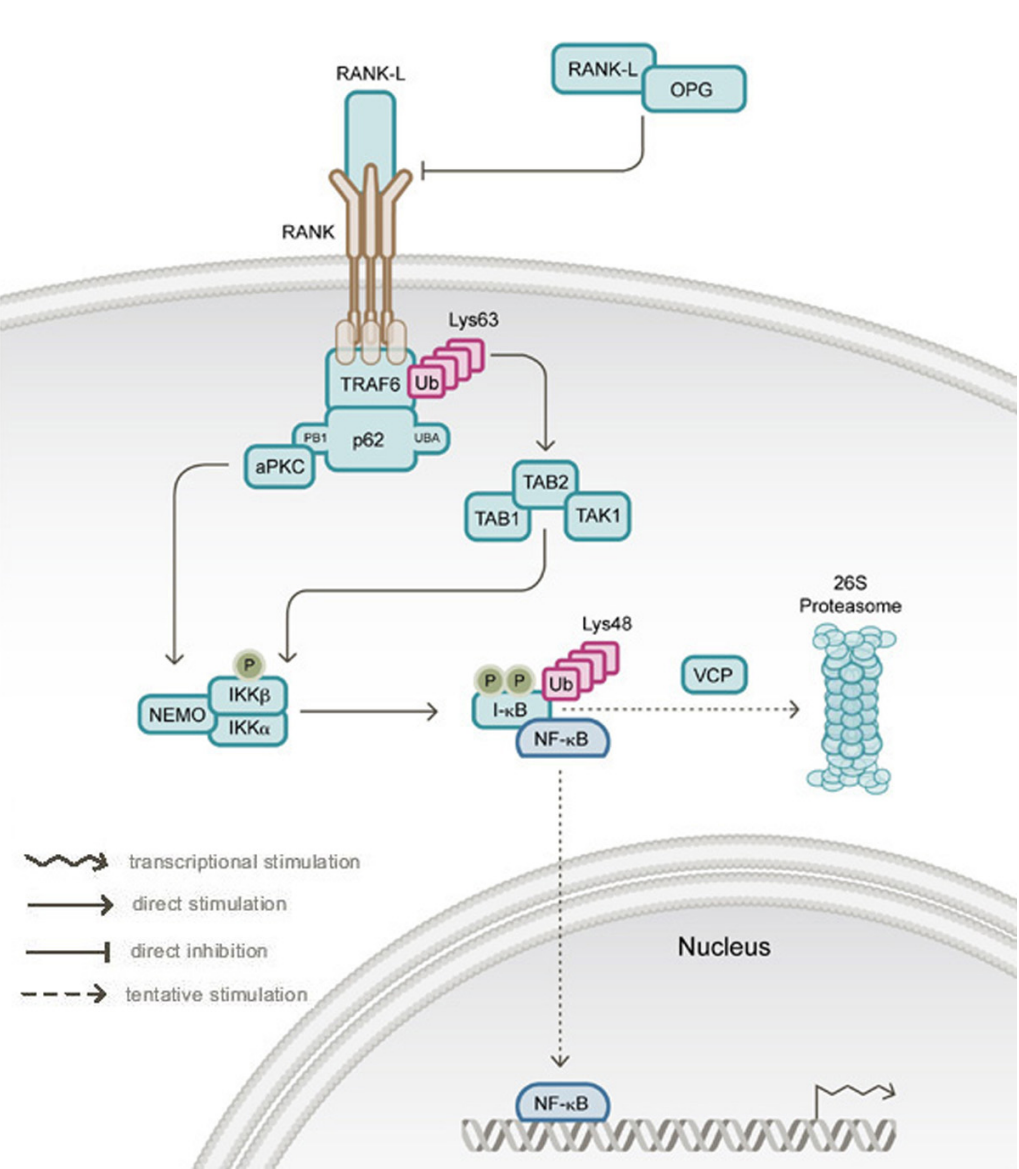
**Schematic overview of the RANK-NF-κB signalling pathway**. The cytokine RANK-L binds to the receptor protein RANK in an interaction antagonised by OPG-RANK-L binding. Upon receptor stimulation by RANK-L, TRAF6 associates with RANK, and the p62 adapter protein binds to TRAF6. Lys63-linked autoubiquitylation of TRAF6 is catalysed by its intrinsic E3 ubiquitin ligase activity (and may be regulated by p62). Through its N-terminal PB1 domain p62 binds aPKC, stimulating the activation of I-κB kinaseß (IKKß). Activation of the TAB1-TAB2-TAK1 complex by binding ubiquitylated TRAF6 also leads to phosphorylation and activation of IKKß. Phosphorylation of I-κB by the activated IKKß complex and subsequent Lys48-linked polyubiquitylation leads to 26S proteasomal degradation of I-κB. This allows NF-κB to enter the nucleus and activate target gene expression. VCP may regulate the proteasomal degradation of I-κB.

Although a slow virus infection of osteoclasts was in the past proposed to be the cause of PDB [6], numerous families have since been documented in which PDB is inherited in an autosomal-dominant fashion [7], establishing a genetic basis for the disease. More recently, it was realised that germline mutations affecting the *Sequestosome 1* (*SQSTM1*) gene, which encodes the p62 signalling adapter protein, commonly occur in PDB patients; these mutations are present in up to 50% of familial and 20% of sporadic cases of PDB [5]. Positional cloning studies have shown that FEO, ESH, and early-onset familial PDB are each caused by different mutations in the receptor activator of NF-κB gene (*RANK*) that all affect the signal peptide of the gene product by introducing amino acid insertions of different lengths [8-10]. RANK is a member of the TNF receptor family [11] that, upon engagement of RANK-ligand (RANK-L), directly interacts with TRAF6, leading to downstream activation of several signalling pathways including an NF-κB pathway that is regulated by p62 ([12]; Figure 1). Homozygous deletions of *OPG*, the gene encoding the soluble RANK-L decoy receptor osteoprotegerin (OPG), have been identified as a cause of juvenile hyperphosphatasia [13,14]. IBMPFD was recently shown to be caused by mutations in the *VCP* (valosin-containing protein, also know as p97) gene [4].

### Signalling pathway affected in PDB and related syndromes

Induction of RANK signalling by RANK-L, a cytokine that is highly expressed in the bone marrow environment, can potentially lead to the activation of AP-1, NFATc1 and NF-κB transcription factors, all of which are important for osteoclastogenesis and/or activity [15]. However, only the signalling pathway resulting in NF-κB activation is thought to involve OPG-RANK and p62 (Figure 1), each of which are (separately) mutated in PDB and related syndromes, indicating that altered function of this particular signalling cascade is likely to be important in PDB aetiology.

Central to several steps within the RANK-NF-κB pathway, as well as many other signalling pathways, is the reversible covalent attachment of ubiquitin (ubiquitylation) to signalling proteins [16]. Upon attachment, ubiquitin acts as a scaffold in protein-protein interactions involving the ubiquitylated target. Ubiquitin can be assembled into polymeric structures or ‘chains’ with different topologies [17] that, in some regards, resemble the different glycan structures within glycoproteins. These different chain topologies result from the way in which ubiquitin attaches; the extreme C-terminal Gly76 residue of ubiquitin becomes linked via an isopeptide bond to an ε-amino group of a Lys side chain in the target protein. In a polyubiquitin chain, any of the seven Lys residues in an individual ubiquitin may form a link with adjacent ubiquitin moieties. Polyubiquitin chains are most commonly linked via Lys48 of ubiquitin, a modification that generally targets the ubiquitylated protein for recognition and degradation by the 26S proteasome complex (the so-called ubiquitin proteasome system, or UPS). By contrast, chains linked via Lys63 generally serve non-degradative roles, such as facilitating protein-protein interactions involved in the formation of multi-protein signalling or DNA repair complexes.

Although temporal aspects of the RANK-NF-κB signalling pathway are not fully established, key steps (some with known dependence upon ubiquitylation) include (see Figure 1): engagement of RANK by RANK-L, which is antagonised by OPG-RANK-L binding; association of TRAF6 with activated RANK; binding of p62 to TRAF6; Lys63-linked autoubiquitylation of TRAF6, catalysed by its intrinsic E3 ubiquitin ligase activity; p62-aPKC-mediated phosphorylation and activation of I-κB kinaseß (IKKß); activation of the TAB1-TAB2-TAK1 complex by ubiquitylated TRAF6, also leading to phosphorylation and activation of IKKß, and phosphorylation of I-κB by the activated IKKß complex and subsequent Lys48-linked polyubiquitylation. These steps result in the 26S proteasomal degradation of I-κB, entry of NF-κB to the nucleus and activation of target gene expression [16].

Whilst RANK and OPG clearly function as upstream components of this signalling pathway, it is presently unclear as to the role(s) played by VCP in osteoclast NF-κB signalling. To date, certain IBMPFD mutations in VCP have been found to disrupt endoplasmic reticulum-associated degradation (ERAD), an ubiquitin-dependent process [18]. Previous studies have shown that VCP can also directly regulate NF-κB signalling in an osteosarcoma (osteoblast-derived) cell line [19], and that VCP binding to ubiquitylated I-κB may promote I-κB degradation [20]. Determining the NF-κB activation status of osteoclasts from IBMPFD patients would, therefore, clearly be most informative. The proposed role of p62 in regulating RANK signalling is discussed in more detail below.

### Disease mechanism(s) in PDB and related syndromes

Consistent with the involvement of RANK-L, OPG, RANK and p62 in an NF-κB signalling pathway that is key to osteoclastogenesis and function, there is increasing evidence to support the hypothesis that disordered RANK-NF-κB signalling may be central to the aetiology of PDB and related syndromes. Firstly, the *RANK* insertion mutations associated with FEO, ESH and early-onset familial PDB are proposed to result in constitutive activation of NF-κB signalling *in vitro* (based on results from cell-based reporter assays) [8-10]. This effect may be due to intracellular retention of the mutant receptors. Secondly, as OPG is a negative regulator of RANK signalling, loss of OPG wild-type function in juvenile hyperphosphatasia is also predicted to promote RANK-NF-κB signalling *in vivo*[13,14]. Finally, two separate groups have shown that under certain conditions, ectopic expression of PDB mutant p62 evokes more efficient activation of NF-κB signalling than the wild-type sequence in cell-based reporter assays [21,22]. In addition, RAW264.7 cells more readily form osteoclast-like cells (OLCs) when transfected with PDB mutant p62 rather than the wild-type. Furthermore, OLCs derived from monocytes from *SQSTM1* mutation-carrying patients (K378X, truncating) showed increased bone resorption *in vitro* when compared with those derived from control monocytes [22]. These observations are again consistent with the activation of NF-κB-dependent responses.

The precise mechanism by which p62 mutations result in disordered RANK-NF-κB signalling *in vivo*, as is suggested by *in vitro* data, is not clear. Certainly, the expression levels of p62 directly influence signalling; depletion of p62 in cultured cells severely inhibits NF-κB signalling [16] and accordingly, genetic inactivation of p62 in mice abrogates NF-κB signalling, resulting in defective osteoclastogenesis when animals are challenged with parathyroid hormone related protein (a bone-resorbing factor; [21]). A recent study noted, however, that in reporter assays, over-expression of wild-type p62 attenuated (rather than potentiated) NF-κB activation when compared with an empty vector control [22]. PDB mutations do not, however, appear to affect p62 protein half-life [22], suggesting that their mechanism of action is unlikely to involve altered protein turnover and expression levels.

In addition, it is noteworthy that evidence to support constitutive NF-κB activation by PDB mutant RANK [8-10] was derived from cell-based reporter assays in which NF-κB activity was higher for the mutant only when protein levels were normalised to that of transfected wild-type RANK (there appeared to be less PDB mutant RANK than wild-type, presumably reflecting their different half-lives). It is questionable whether this normalisation was appropriate; indeed a recent study [23] indicated that ‘physiological’ expression (i.e. not over-expression) of mutant RANK in stably-transfected cell lines did not result in constitutive activation of NF-κB.

Notably, all 12 of the separate *SQSTM1* mutations identified to date cluster in and around the C-terminal ubiquitin-associated (UBA) domain of p62 [24]. The UBA domain mediates the ubiquitin binding properties of the protein. These mutations include five truncating mutations that delete most or all of the UBA domain, and seven missense mutations located within the UBA domain [22,25-30]. Functional studies using protein binding assays show that all of the p62 PDB mutations manifest as loss of ubiquitin binding *in vitro*[31,32], indicating that the disease mechanism is likely to involve the inability of mutant p62 to establish regulated protein-protein interactions with an ubiquitylated osteoclast protein(s). Interestingly, the *VCP* mutations found in IBMPFD patients also cluster in the N-terminal ubiquitin binding region of the protein [4]. It is unclear, however, whether they similarly affect ubiquitin binding, perhaps to overlapping p62 substrates. Preliminary genotype/phenotype analyses support the significance of p62-mediated ubiquitin binding in osteoclast homeostasis and further verify the existence of a causal relationship between p62 mutations and PDB [33]. Several observations suggest that PDB severity (e.g. decreased age at diagnosis, increased number of bones affected) increases with the severity of the effects of different mutations on p62-ubiquitin binding. For example, the p62 truncating mutations, which remove most or all of the UBA domain, are the most detrimental to p62-ubiquitin binding function *in vitro*, resulting in a more severe phenotype than the missense mutations [30,22]. Moreover, the missense mutation that was found to be the least detrimental in *in vitro* p62-ubiquitin binding assays (P387L) appears to produce a relatively mild phenotype in affected individuals [29].

It is also interesting to note that similar p62 germline mutations appear to account for both monostotic and polyostotic forms of PDB (in the latter, non-contiguous skeletal segments are affected), suggesting that additional local factors (e.g. mechanical loading, trauma etc.) may in some cases act as a disease trigger.

Key questions to resolve include what the ‘normal’ ubiquitylated targets of p62 in osteoclasts are and how loss of ubiquitin binding by p62 affects RANK-NF-κB signalling and ultimately leads to PDB. One of the proposed functions of p62 (which is in fact multi-functional [16]) is to regulate NF-κB signalling in response to nerve growth factor (NGF), by controlling the Lys63-linked polyubiquitylation of TRAF6 [34]. Whether p62 performs a similar role in RANK signalling awaits confirmation, although interestingly, p62-mediated ubiquitylation of TRAF6 in response to NFG appears to require the ubiquitin binding activity of p62 [34]. This observation is somewhat at variance with the finding that a p62 construct carrying the most common P392L PDB missense mutation, and which causes loss of ubiquitin binding of p62 [31], apparently evokes more efficient activation of NF-κB signalling in reporter assays than wild-type p62 [21]. However, a more recent study also using reporter assays noted that although PDB mutant p62 constructs activated NF-κB signalling more efficiently than wild-type, compared with an empty vector control, all p62 constructs showed reduced activation, suggesting the mutations may actually diminish a repressive function of p62 with respect to RANK-NF-κB signalling [22]. To what extent these discrepancies represent differences in experimental protocols and perhaps the artificial effect of protein over-expression remains to be seen, but clearly further studies are required to precisely clarify the effects of p62 (and indeed RANK) mutations on NF-κB signalling in more physiologically relevant settings.

## Disease models, knockouts and assays

The various cell-based reporter assays and *in vitro* models for differentiating cells (e.g. RAW264.7 or monocytes) carrying PDB mutant p62 into OLCs [21,22] represent potential assay systems for developing high-throughput screens for the identification of lead compounds which may be useful in correcting disordered osteoclast NF-κB signalling. Although p62 null mice exist, in the context of PDB, they simply serve to confirm the important role(s) of p62 in osteoclastogenesis; the community awaits with anticipation the generation of animal models carrying p62 PDB mutations, and their subsequent phenotypic characterisation.

## Disease targets and ligands

Current treatments for PDB are mainly limited to anti-resorptive therapy using bisphosphonates, which selectively target and induce apoptosis of osteoclasts via a mechanism thought to involve inhibition of protein prenylation [35].

If the disordered RANK-NF-κB signalling inferred from the *in vitro* studies of PDB mutant p62 (and mutant forms of other components in the RANK-NF-κB signalling axis) is indeed confirmed to be a reflection of disease-related events *in vivo*, the disease aetiology in PDB is likely to involve abnormal sensitivity of osteoclast RANK-NF-κB signalling in response to cytokines (and 1,25-dihydroxyvitamin D3) [36]. It also seems reasonable that disordered NF-κB signalling, arising via a different mechanism, could similarly underlie the disease mechanism in sporadic PDB as phenotypically sporadic and familial PDB are very similar.

Several strategies, based upon manipulation of osteoclast NF-κB signalling, are under development or envisaged to treat PDB syndromes. For example, hormones, drugs or antibodies could be used (or have been applied) to regulate the expression of RANK-L and OPG, or binding of RANK-L to RANK (reviewed in [37] and discussed in [38]). For example, Amgen have developed a monoclonal antibody to RANKL (AMG162) which is currently in clinical trials for various bone diseases (see [39] and references therein); previously they also developed an OPG fusion protein (OPGfc) which was subsequently withdrawn from clinical practice. Additionally, recombinant OPG has been used to treat a patient with juvenile PDB [40].

Alternatively, RNA silencing *in vivo* could be used to neutralise mutant p62 transcripts, as attempted in Huntington's disease [41]. Finally, directly interfering with the p62-TRAF6 interaction using a short peptide equivalent to the TRAF-binding domain sequence of p62, offers the possibility of selectively targeting TRAF6-mediated NF-κB signalling without affecting AP-1- or NFATc1-mediated processes. In the case of NGF signalling (which also depends upon TRAF6 and p62), this approach was recently used in cultured cells to inhibit NGF-induced neurite outgrowth, a process dependent on TRAF6-p62-mediated NF-κB signalling [42]. The caveat to all of these approaches is achieving osteoclast-specific effects, although combining any potential compound with a vehicle or carrier which has a high affinity for bone matrix (a property which underlies the apparent selectivity of the bisphosphonates) may prove to be a useful delivery system.

## New frontiers in drug discovery

Modulation of the osteoclast RANK-NF-κB signalling axis, and its control by ubiquitylation, may represent a viable therapeutic strategy for the treatment of PDB syndromes as well as other diseases where excessive bone resorption or remodelling is a feature, including osteoporosis, peridontal disease and rheumatoid arthritis. Indeed, the demonstration that inhibition of NF-κB signalling (using a cell-permeable peptide inhibitor of the IKKß complex) blocks osteoclastogenesis and prevents *in vivo* inflammatory bone destruction [43] is entirely supportive of this concept. A more complete and precise description of the molecular events that underlie RANK-NF-κB signalling in osteoclasts is clearly a next step towards this important goal.

## Abbreviations

PDB, Paget's disease of bone; FEO, familial expansile osteolysis; ESH, expansile skeletal hyperphosphatasia; IBMPFD, inclusion body myopathy associated with PDB and frontotemporal dementia; SQSTM1, Sequestosome 1; RANK, receptor activator of NF-κB; RANK-L, RANK-ligand; OPG, osteoprotogerin; VCP, valosin-containing protein; UPS, ubiquitin proteasome system; IKKß, I-κB kinaseß; ERAD, endoplasmic reticulum-associated degradation; OLC, osteoclast-like cell; UBA, ubiquitin-associated (domain).

## Competing interests

The authors declare that they have no competing interests.

## Publication history

Republished from Current BioData's Targeted Proteins database (TPdb; ).
